# On the use of exposure therapy in the treatment of anxiety disorders: a survey among cognitive behavioural therapists in the Netherlands

**DOI:** 10.1186/s40359-015-0083-2

**Published:** 2015-08-05

**Authors:** David Sars, Agnes van Minnen

**Affiliations:** Dutch Association for Behavioural and Cognitive Therapy (VGCt), Utrecht, The Netherlands; UvA Minds You, Academic Training Centre, Amsterdam, The Netherlands; Mettaminds, Mindfulness based projects, Amsterdam, The Netherlands; Overwaal, Centre for Anxiety Disorders, Pro Persona, Nijmegen, The Netherlands; Radboud University, Behavioural Science Institute, NijCare, Nijmegen, The Netherlands

**Keywords:** Exposure therapy, Cognitive therapy, Behavioural therapy, Education, Dissemination, Empirically supported treatment, Social anxiety disorder, Obsessive compulsive disorder, Phobia, Panic Disorder (with or without agoraphobia)

## Abstract

**Background:**

Although research has shown exposure therapy to have earned its rank among empirically supported treatments (ESTs) for anxiety disorders, several US-based studies suggest it to be underused in clinical practice. Data on exposure use in Europe is mainly lacking, whereas its state of dissemination in countries such as the Netherlands has remained uncharted. Therefore, this study examined the use of exposure therapy among members of the Dutch Association for Behavioural and Cognitive Therapy (*VGCt*), as well as explored therapist, educational and contextual variables that could facilitate its dissemination in clinical practice.

**Methods:**

Respondents (*n* = 490) were surveyed on clinical interventions used in their treatment for social anxiety disorder, phobia, OCD and panic disorder. Data was collected on the use of (disorder) specific interventions, therapists’ attitudes on exposure, treatment experience, current educational status, educational background and workplace characteristics.

**Results:**

Analysis of the data showed that most therapists implemented exposure frequently, but that exposure use still warrants improvement, specifically for certain (disorder-specific) interventions that were accordingly underused. Confirming our hypothesis, we found that clinicians who practiced exposure regularly also reported a greater willingness to use the treatment, perceived the method as more credible, and saw fewer barriers for its usage than those who did so less. The use of (disorder-) specific interventions, such as in vivo exposure (therapist as well as self-directed), exposure and response prevention for OCD, and interoceptive exposure for panic disorder, was positively related to level of education. While most were satisfied with the training they had received, therapists did report a need for additional instruction in targeted practical, empirical, and diagnostic skills.

**Conclusions:**

Our findings support the conclusion that the dissemination of exposure therapy in the Netherlands progresses well, but that education in certain (disorder-specific) techniques merits augmentation. To bridge the gap between research and clinical practice, future research should therefore focus on new, preferably blended approaches to training clinicians in exposure techniques.

## Background

Cognitive Behavioural Therapy (CBT), with exposure therapy as its principal modality, takes a prominent place in international guidelines for the treatment of anxiety disorders (e.g. National Institute for Health and Clinical Excellence 2011; LSMR - Dutch National Steering-Group Multidisciplinary Guideline Development for Mental Healthcare 2013). These guidelines are based on extensive empirical support to suggest that exposure therapy is effective in the treatment of social anxiety disorder (Fedoroff & Taylor 2001; Feske & Chambless 1995), (specific) phobia (Wolitzky-Taylora et al. 2008; Craske 1999), obsessive compulsive disorder (OCD; Rosa-Alcázar et al. 2008; Abramowitz 1996), panic disorder with or without agoraphobia (Sánchez-Meca et al. 2010; Van Balkom et al. 1997), posttraumatic stress disorder (PTSD; Cahill et al. 2009; Bradley et al. 2005), and generalized anxiety disorder (Bradley et al. 2005; Gould et al. 1997).

Yet, despite the empirical evidence of its efficacy, the gap between theory and practice has remained, with exposure-based interventions still being underused in clinical practice. A US survey of 500 psychologists found that, although 71 % reported having a cognitive behavioural orientation, 26 % seldom or never used exposure and response prevention for OCD, 76 % seldom or never used interoceptive exposure for panic disorder, while less than one third reported implementing exposure techniques for social anxiety on a regular basis (Freiheit et al. 2004). Another US-based study found that 83 % of therapists seldom or never used imaginal exposure for PTSD (Becker et al. 2004). Furthermore, two patient surveys established that a minority (around 20 %) of patients reported receiving exposure therapy for their anxiety disorder (Marcks et al. 2009; Goisman et al. 1999). In sum, the dissemination of exposure therapy merits improvement.

However, because most of these studies took place in the US, data on exposure usage in Europe is mainly lacking. One study that was conducted among German psychotherapists was in line with findings in the US that exposure is underused and reported that more than half of the therapists did not use exposure for OCD (Külz et al. 2010). To fill the gap in research in this area between the US and Europe, the present study examines the extent to which Dutch therapists with a cognitive behavioural orientation apply exposure, focusing on the treatment of the four most prevalent anxiety disorders: social anxiety disorder, (specific) phobia, OCD and panic disorder (with or without agoraphobia).

To chart the state of the art on exposure dissemination more exhaustively, we wished to gain insight into the reasons why mental health professionals do or do not use exposure by including questions on their training and professional attitudes about exposure treatments. In previous studies, for instance, therapists gave deficiency or absence of *specialized* training as the main reason for not using exposure-based therapies (Külz et al. 2010; Weissman et al. 2006; Becker et al. 2004). In our current survey we hence paid special attention to the type of exposure training therapists had received and the extent to which this was considered satisfactory. As attitudes and beliefs have been shown to play a considerable role, we were curious to know whether and to what extent exposure therapy invited approval or rejection, given its allegedly invasive nature. Studies have found clinicians to harbour negative notions, with exposure being deemed ‘insensitive’, ‘rigid’, ‘ineffective’, ‘potentially iatrogenic’, ‘not generalizable to the real world’, and even ‘unethical’ (Olatunji et al. 2009; Richard & Gloster 2007; Feeny et al. 2003). Importantly, an earlier study on motivational factors for therapists to treat PTSD-patients with exposure, found that therapists used more exposure as they valued exposure more credible and perceived fewer barriers for its usage (e.g. fear of symptom exacerbation and dropout; van Minnen et al. 2010).

With our Internet-based survey among cognitive behavioural therapists we sought answers to the following three questions: (a) To what extent do Dutch therapists apply exposure therapies in their treatment of anxiety disorders compared to their US colleagues?; (b) Which attitudes about exposure influence its usage?; and (c) What is the relationship between training, treatment experience and the use of exposure? We predicted that (a) compared to their US colleagues Dutch therapists would use exposure more frequently, that (b) the therapists that use exposure more frequently see fewer barriers for its usage and perceive the method as more credible, and (c) have received more (comprehensive) training and are more experienced than their peers who practice exposure less often.

## Methods

### Participants and procedure

We approached 3085 members of the Dutch Association for Behavioural and Cognitive Therapists (*VGCt*), whose status was further defined as ‘therapists in training’, i.e. psychologists with a postgraduate degree (MA, MSc, or PhD) in clinical psychology receiving training in CBT, ‘certified therapists’, i.e. clinical psychologists licensed and practicing as cognitive behavioural therapists, and ‘supervisors’, i.e. experienced clinical psychologists and therapists providing training in CBT. In December 2010 they were sent an invitation by e-mail, together with a link to our survey. By following this link, respondents were presented our policy statement on confidentiality, i.e. that their responses would be stored and processed anonymously, after which they were given the choice to proceed. In accordance with the Dutch code of conduct for scientific practice no additional ethics approval was sought, as this present study involved a onetime survey only, without manipulations or emotional burden for the respondents. Furthermore, following the procedure adopted by Freiheit et al. (2004), we minimized response bias by avoiding characterizing exposure therapies as being ‘empirically supported’ as much as possible throughout the survey.

The dataset of the 893 members that returned the survey (response rate = 28.9 %) was checked for data conversion errors (survey data to SPSS), outliers, and missing data (*n* = 30). Respondents who had never or rarely treated patients with anxiety disorders (0-10 % of their caseload) in the past 12 months (*n* = 79 and *n* = 294, respectively), were redirected to the end of the survey. The final sample for analysis consisted of 490 respondents of whom 153 (31.2 %) were therapists in training (mean age 37.3 years; *SD* = 8.4), 190 (38.8 %) certified therapists (mean age 46.0 years; *SD* = 10.3), and 147 (30.1 %) supervisors (mean age 53.4 years; *SD* = 7.9). Of this sample the average age of respondents was 45.6 year (*SD* = 11.1), with the greater majority being female (75.3 %). Most respondents (59.4 %) worked in secondary healthcare (e.g., general hospitals and mental health facilities), for which in the Netherlands a referral from a primary care physician is required; 24.7 % worked in a private or group practice treating both referred and non-referred patients, while 5.5 % held (usually small) practices taking patients without referral. The distribution of status, age, sex and registration in our sample corresponded with the distribution in the *VGCt* membership register (2010), indicating a representative sample.

### Outcome measures

#### The use of exposure

Respondents were asked if they applied exposure therapies (Yes/No) and to select from a number of options the two main reasons why they did or did not do so. If yes, respondents were asked to indicate whether they (had) treated social anxiety, (specific) phobia, OCD and panic disorder and subsequently directed to a subset of questions where they could indicate for each of the disorders how often they applied a certain intervention on a 4-point frequency scale (1 = Never; 4 = Frequently). The choice of interventions was based on the national multidisciplinary anxiety disorders guidelines (LSMR - Dutch National Steering-Group Multidisciplinary Guideline Development for Mental Healthcare 2009) and recent research literature. The items specified basic treatment components, such as explaining the rationale of exposure, and specific interventions, such as in vivo exposure. Because the Dutch guidelines also mention other interventions (e.g. cognitive skill training and general techniques such as breathing exercises), these were added to the list as well.

#### Attitudes toward exposure

Items of the ‘Willingness’, ‘Treatment Credibility’ and ‘Perceived Barriers’ scales were modified from an earlier study by van Minnen et al. (2010), and were scored on an 8-point disagree-agree Likert scale, with higher scores reflecting higher values for the relevant attitude. Total scale scores were calculated by averaging the scales’ item scores.

#### Willingness

This scale measures the degree to which the therapist is willing to apply exposure techniques and consists of 11 items (e.g., ‘Would I actually use exposure during a session?’; Cronbach’s α = 0.91).

#### Treatment credibility

The four items in this scale assess the respondent’s stance on the credibility of exposure as an intervention (e.g., ‘If a good friend were to have an anxiety disorder, I’d advise exposure as a treatment option’; Cronbach’s α = 0.85).

#### Perceived barriers

The scale gauges the clinician’s perceived barriers for using exposure and comprises the following three subscales:

#### Personal preference

This 5-item scale measures the degree to which the respondent has an affinity with exposure (e.g., ‘I read a lot about exposure’; Cronbach’s α = 0.86).

#### Avoidance

This 10-item scale measures the extent to which respondents fearfully avoid the use of exposure (e.g., ‘I don’t dare to practice exposure exercises with my clients’; Cronbach’s α = 0.87).

#### Practical limitations

These 2 items examined which resources are available at the respondent’s workplace for the practice of exposure therapies, among which typical tools such as treatment protocols and stimulus or other supporting material.

#### Training and experience

With this 6-item scale we gauged the extent to which respondents were trained in the practice of exposure (e.g., ‘I am fully informed of the most recent developments concerning exposure treatments’; Cronbach’s α = 0.88). Items were scored on an 8-point disagree-agree Likert scale, with higher scores representing higher levels of training. Respondents were also asked to indicate their total treatment experience (in years) and actual caseload in terms of the number of patients with an anxiety disorder they had treated relative to their overall caseload.

Next, for each of the four anxiety disorders respondents were instructed to specify exposure training in terms of practical, diagnostic and empirical skills learned on an 8-point Likert scale (1 = None; 8 = Comprehensive).

### Analysis

Associations between the use of exposure, attitudes towards exposure, and training and experience were calculated using Spearman rank correlations (ρ). To correct for multiple comparisons an alpha of 0.001 was adopted.

## Results

### Use of exposure

Almost all respondents (97.8 %) reported using exposure for the treatment of anxiety disorders and gave as the main rationale ‘exposure is empirically supported’ and ‘personal clinical experience suggests it is effective’. Table 1 gives an overview of the frequency and type of exposure interventions the therapists applied for the four anxiety disorders.

**Table 1:** Overview of interventions used (in percentages) by Dutch cognitive behavioural therapists in the treatment of anxiety disorders

	Social Anxiety (*n* = 476)				(Specific) Phobia (*n* = 448)				OCD (*n* = 443)				Panic (*n* = 467)			
	Frequently	Occasionally	Sometimes	Never	Frequently	Occasionally	Sometimes	Never	Frequently	Occasionally	Sometimes	Never	Frequently	Occasionally	Sometimes	Never
*Basic interventions*																
Drawing-up anxiety hierarchy	69.9	22.1	6.5	1.5	82.8	10.3	4.2	2.7	75.3	15.6	6.8	2.3	83.2	11.3	3.4	2.1
Explaining rational exposure	94.5	4.2	1.1	0.2	96.3	2.9	0.4	0.4	94.3	4.1	1.1	0.5	96.4	3.0	0.4	0.2
*Exposure interventions*																
Therapist-directed in vivo exposure	35.1	37.2	21.2	6.5	52.2	30.8	12.1	4.9	39.1	38.1	17.4	5.4	52.3	26.1	16.7	4.9
In vivo self-exposure	78.4	14.9	4.8	1.9	79.9	13.6	4.5	2.0	82.1	10.6	5	2.3	82.7	11.6	3.6	2.1
Imaginal exposure	24.6	37.8	28.4	9.2	26.8	37.7	23.0	12.5	20.5	34.6	28.4	16.5	28.1	31.6	24	16.3
Exposure and response prevention	45.4	28.8	15.5	10.3	47.6	23.4	16.1	12.9	87.4	9.9	2.0	0.7	47.3	20.6	17.1	15
Interoceptive exposure	13.7	26.3	33.1	26.9	7.8	26.1	29.2	36.9	7.4	19.6	29.8	43.2	61	16.9	13.7	8.4
Exposure homework assignments	89.1	8.4	1.9	0.6	89.2	8.3	1.8	0.7	89.2	8.8	1.1	0.9	90.7	6.9	1.5	0.9
*CT*																
Cognitive restructuring	83.8	14.1	1.9	0.2	67.4	22.1	8.5	2.0	76.3	17.6	4.5	1.6	82.9	14.3	2.6	0.2
Homework assignments for cognitive restructuring	74.4	19.3	5.7	0.6	59.4	23.9	11.6	5.1	68.6	22.1	7.0	2.3	76.8	17.8	4.5	0.9
*General*																
Psycho-education	89.5	8.0	1.9	0.6	85.7	7.8	2.9	3.6	88.5	6.1	2.0	3.4	89.1	6.6	1.9	2.4
Breathing exercises	29.8	30.5	22.3	17.4	25.9	29	21.4	23.7	16.7	24.8	23.9	34.6	43.7	25.9	14.3	16.1
Relaxation exercises	28.2	35.9	25.4	10.5	26.6	33.2	23	17.2	19.9	29.3	26	24.8	44.5	29.6	14.8	11.1

#### Social anxiety disorder

The exposure interventions the respondents applied most frequently for this disorder were ‘exposure-based homework assignments’ (89.1 %), ‘in vivo self-exposure (i.e., practiced by the patient between sessions; 78.4 %), and ‘exposure and response prevention’ (45.4 %).

#### Specific phobia

The most frequently used exposure techniques for specific phobia were ‘exposure-based homework assignments’ (89.2 %), ‘in vivo self-exposure’ (79.9 %), and ‘therapist-directed in vivo exposure’ (i.e., practiced together with the therapist during sessions; 52.2 %).

#### Obsessive compulsive disorder (OCD)

For OCD the therapists reported applying ‘exposure-based homework assignments’ (89.2 %), ‘exposure and response prevention’ (87.4 %), and ‘in vivo self-exposure’ (82.1 %) the most regularly.

#### Panic disorder

Here also ‘exposure-based homework assignments’ was the most frequently implemented intervention (90.7 %), followed by ‘in vivo self-exposure’ (82.7 %), and ‘interoceptive exposure’ (61 %).

#### Other interventions

Other cognitive interventions frequently used alongside exposure techniques were ‘cognitive restructuring’ (range 67.4 % - 83.8 %) and ‘general psycho-education’ (85.7 % - 89.5 %). Breathing and relaxation exercises were used relatively little (16.7 % - 44.5 %).

### Attitudes toward exposure

#### Willingness

The mean score for all respondents (*n* = 490) was 6.25 (*SD* = 1.26; sample range 4.55 – 7.73), reflecting an overall favourable stance toward the use of exposure therapies.

#### Treatment credibility

The mean score of 7.16 on this scale (*SD* = 0.98; sample range 1.00 – 8.00) indicates that our respondents deemed exposure therapies very credible.

### Perceived barriers

#### Personal preference

With a mean score of 6.02 (*SD* = 1.30; sample range 1.00 – 7.00) exposure therapy was generally considered to be an attractive treatment option.

#### Avoidance

The mean score on this scale was 2.05 (*SD* = 0.89; sample range 1.00 – 7.00), indicating that relatively few respondents avoided exposure therapy.

#### Practical limitations

55.3 % of the respondents were not satisfied with the exposure resources at their workplace in terms of lack of proper protocols, while 22.2 % also reported an insufficient availability of materials supporting the practice of exposure, such as recording equipment, film material, certain animals and sounds.

### Associations between attitudes and usage

Our correlation analyses of the respondents’ attitudes toward and the practice of exposure revealed a consistent pattern. The willingness, treatment credibility and personal preference scale scores correlated positively with the frequency of use of in vivo exposure (therapist and self-directed) and exposure-based homework assignments. Table 2 lists all Spearman correlations. The scores for the three scales also showed a positive correlation with the use of disorder-specific interventions, such as exposure and response prevention for OCD, and interoceptive exposure for panic disorder. The extent of practical limitations correlated negatively to the use of therapist-directed in vivo exposure only. Correlations with the avoidance scale were not significant.

**Table 2:** Correlations (Spearman’s rho) for exposure use and exposure attitude scale scores

	Willingness	Credibility	Avoidance	Personal preference	Practical limitations
*Social Anxiety*					
Therapist-directed in vivo exposure	.34^a^	.18^a^	-.12	.25^a^	-.18^a^
In vivo self-exposure	.22^a^	.25^a^	-.06	.24^a^	-.02
Imaginal exposure	.03	-.06	-.05	.01	-.07
Exposure and response prevention	.08	.08	.01	.07	-.04
Interoceptive exposure	.08	-.40	.00	.00	-.01
Exposure-based homework assignments	.25^a^	.28^a^	-.10	.31^a^	-.02
*(Specific) Phobia*					
Therapist-directed in vivo exposure	.37^a^	.20^a^	-.15	.24^a^	-.20^a^
In vivo self-exposure	.17^a^	.27^a^	-.16^a^	.22^a^	-.12
Imaginal exposure	.03	.00	-.09	-.01	-.08
Exposure and response prevention	.10	.12	.00	.09	-.10
Interoceptive exposure	.10	.01	-.07	.03	-.09
Exposure-based homework assignments	.18^a^	.24^a^	-.17^a^	.26^a^	.12
*OCD*					
Therapist-directed in vivo exposure	.29^a^	.20^a^	-.16^a^	.23^a^	-.16^a^
In vivo self-exposure	.12	.17^a^	-.11	.18^a^	-.07
Imaginal exposure	.04	.00	-.08	-.02	-.09
Exposure and response prevention	.15^a^	.26^a^	-.13	.24^a^	-.10
Interoceptive exposure	.04	-.03	-.05	.00	-.05
Exposure-based homework assignments	.16^a^	.28^a^	-.13	.23^a^	-.09
*Panic*					
Therapist-directed in vivo exposure	.30^a^	.25^a^	-.15^a^	.25^a^	-.15^a^
In vivo self-exposure	.17^a^	.23^a^	-.14	.22^a^	-.05
Imaginal exposure	-.02	-.09	.02	-.04	-.01
Exposure and response prevention	-.01	.05	-.05	-.01	-.07
Interoceptive exposure	.27^a^	.33^a^	-.14	.21^a^	-.06
Exposure-based homework assignments	.22^a^	.26^a^	-.08	.25^a^	-.02

^a^Significant at α = 0,001 (two-sided)

### Training

Almost all therapists reported having experience in treating patients with social anxiety disorders (97.1 %), with comparable percentages for panic disorder (95.3 %), specific phobia (91.4 %), and OCD (90.4 %); mean experience was 16.1 years (*SD* = 9.44). An average of 12.3 (*SD* = 10.0) patients in their current caseload was being treated for anxiety disorders, and 14.9 (*SD* = 11.8) patients in the last three months. The number of sessions for successful treatment was estimated at around 15.3 (*SD* = 6.0).

With a total score of 6.45 on the training scale (*SD* = 1.26; sample range 1.00 – 8.00), the respondents rated themselves as being sufficiently to well trained in exposure therapies. Post-hoc analysis revealed a significant difference for therapist status (*F* (2.487) = 20.61, *p* = 0.001), where, as expected, therapists in training had indicated to feel the least and supervisors the most confident in practicing exposure.

In general, most respondents (64.1 %) reported having received a sufficient degree of postgraduate training in exposure: 25.6 % reported having received CBT training with limited attention to exposure, 24.1 % clinical supervision from an experienced professional, 20.7 % basic practical skills training and clinical experience, 17.9 % workshop education, and 11.7 % dedicated training in exposure therapy. Finally, although most were content with their exposure education, 55.6 % of the therapists in training, 35.8 % of certified therapists, and 23.1 % of the supervisors expressed a need for more exposure-specific instruction.

### Disorder-specific training

Table 3 shows the respondents’ mean scores for the exposure training they received in terms of practical, diagnostic and empirical skills for each type of anxiety disorder. We found no significant differences in therapist status, except for training in practical (*F* (2, 10.43) = 5.67, *p* < .004) and diagnostic skills for OCD (*F* (2, 11.53) = 6.89, *p* < .001), where supervisors had received significantly more instruction and training than therapists in training.

**Table 3:** Mean score for type of training received per disorder

	Social anxiety	Specific phobia	OCD	Panic
Practical skills	5.16	5.32	5.02	5.48
Diagnostic skills	4.83	5.18	5.01	5.31
Empirical skills	4.98	5.26	5.03	5.37

Note: The scale runs from 1 (none) to 8 (very much) with 5 reflecting sufficient training

### Associations between training and exposure use

Table 4 presents all Spearman correlations for type of training received and the use of exposure interventions. Overall, the extent of exposure training (practical, diagnostic and empirical) consistently correlated positively with the use of in vivo exposure (therapist and self-directed) and the use of exposure-based homework assignments. Received education also correlated positively with disorder-specific exposure interventions (e.g., exposure and response prevention for OCD, and interoceptive exposure for panic disorder).

**Table 4:** Correlations (Spearman’s rho) for exposure use and measures of type of education received per disorder

	Social Anxiety (*n* = 476)			(Specific) Phobia (*n* = 448)			OCD (*n* = 443)			Panic (*n* = 467)		
	Practical	Diagnostic	Empirical	Practical	Diagnostic	Empirical	Practical	Diagnostic	Empirical	Practical	Diagnostic	Empirical
*Exposure interventions*												
Therapist-directed in vivo exposure	.18^a^	.17^a^	.12^a^	.15^a^	.14	.14^a^	.24^a^	.19^a^	.21^a^	.21^a^	.19^a^	.16^a^
In vivo self-exposure	.20^a^	.16^a^	.21^a^	.16^a^	.15^a^	.16^a^	.29^a^	.27^a^	.21^a^	.25^a^	.22^a^	.21^a^
Imaginal exposure	.02	.05	.01	.08	.09	.06	.06	.06	.02	.04	.02	.02
Exposure and response prevention	.08	.13	.09	.11	.10	.13	.30^a^	.28^a^	.25^a^	.11	.09	.11
Interoceptive exposure	-.01	.06	.04	.14	.12	.12	.05	.05	.04	.32^a^	.31^a^	.26^a^
Exposure-based homework assignments	.20^a^	.15^a^	.21^a^	.19^a^	.17^a^	.18^a^	.30^a^	.29^a^	.26^a^	.29^a^	.27^a^	.24^a^

^a^Significant at α = 0,001 (two-sided)

### Associations for training, experience and caseload with attitudes and intervention use

We next examined training, treatment experience and caseload in relation to attitudes about exposure; see Table 5 for all corresponding Spearman correlations. The results are consistent with our expectation that more extensive training in exposure correlates positively with more positive attitudes toward the method. Notably, neither treatment experience nor caseload correlated significantly with attitudes toward exposure.

**Table 5:** Correlations (Spearman’s rho) between exposure attitude scale scores and training, experience and caseload

	Training	Experience	Caseload
Willingness	.20^a^	-.10	.05
Credibility	.25^a^	-.08	.12
Personal preference	.32^a^	.07	.08
Avoidance	-.22^a^	-.14	-.13
Practical limitations	.25^a^	-.05	-.06

^a^Significant at α = 0,001 (two-sided)

However, treatment experience and caseload did correlate significantly with the use of specific exposure interventions (see Table 6). Our analysis yielded positive correlations for caseload and the use of in vivo exposure (therapist and self-directed) for nearly all disorders, as well as for years of experience and the use of disorder-specific exposure interventions, such as exposure and response prevention for OCD and imaginal exposure for all anxiety disorders.

**Table 6:** Correlations (Spearman’s rho) for exposure techniques applied, experience and caseload for each of the four anxiety disorders

	Social anxiety (*n* = 476)		(Specific) Phobia (*n* = 448)		OCD (*n* = 443)		Panic (*n* = 467)	
	Experience	Caseload	Experience	Caseload	Experience	Caseload	Experience	Caseload
*Exposure interventions*								
Therapist-directed in vivo exposure	.04	.14^a^	.09	.18^a^	.11	.21^a^	.10	.12
In vivo self-exposure	.04	.14^a^	.12	.17^a^	.14	.13	.13	.13
Imaginal exposure	.21^a^	-.06	.21^a^	.01	.18^a^	.05	.17^a^	-.05
Exposure and response prevention	.08	.03	.10	.02	.16^a^	.13	.10	.01
Interoceptive exposure	.12	.04	.17^a^	.01	.13	.02	-.03	.12
Exposure-based homework assignments	.06	.07	.14	.12	.13	.11	.10	.08

^a^Significant at α = .001 (two-sided)

## Discussion

With our survey we sought to establish the current usage of exposure techniques for the treatment of anxiety disorders in the Netherlands. The results showed that the vast majority of the cognitive behavioural therapists who responded to our invitation (97.8 %; *n* = 450) used some form of exposure therapy in their treatment of patients with social anxiety, (specific) phobia, OCD, and panic disorder. As the main reasons for doing so they stated considering exposure interventions to be effective and empirically supported. Exposure was further viewed as a credible and attractive treatment option and the respondents saw few barriers for its usage. Of all techniques, exposure-based homework assignments were applied most frequently for all four anxiety disorders, closely followed by in vivo self-exposure. Interestingly, exposure was thus mostly practiced outside the formal therapy sessions.

Compared to the rates Freiheit et al. (2004) reported for the US, our data suggests that in the Netherlands patients with anxiety disorders far more frequently receive exposure-based treatments. Looking at disorder-specific interventions, in the US 26 % of OCD patients did not receive exposure or response prevention, compared to only 2.7 % in the Netherlands. Also, 76 % of US patients with panic disorder were not treated with interoceptive exposure, versus 22.1 % of Dutch patients. These large discrepancies may be due to the fact Freiheit et al. (2004) did not restrict their survey to cognitive behavioural therapists as we did, and that there is 7 years between the two studies. With regard to the latter, more recent studies in the US showed more use of exposure: 65 % used interoceptive exposure for panic disorder (Wolf & Goldfried 2014), and 88.4 % used in-session exposure to social situations for social anxiety disorder (McAleavy et al. 2014). Further, the Freiheit study used a more neutral title for their survey (“Treatment of Anxiety Disorders”), whereas we clearly stated in our invitation that the survey concerned exposure therapy. Therefore, our recruitment procedure may have caused a selection bias by mainly attracting therapists with a special interest in exposure treatment. Also, CBT is a dominant therapy in the Netherlands, where many clinical psychologists receive dedicated training in CBT, including exposure techniques. Accordingly, the Dutch Association for Behavioural and Cognitive Therapists (VGCt) has more than 3500 members. With around 4500, its US equivalent, the ABCT, has proportionally far fewer members.

Our survey did demonstrate that, in general, Dutch therapists have a positive attitude toward exposure therapy, deeming it a reliable and viable treatment option. In line with Shafran et al. (2009), we showed that a positive attitude significantly relates to usage, with respondents that practiced exposure on a regular basis also reporting a greater affinity with and willingness to apply the various exposure techniques for the four anxiety disorders we evaluated, as well as disorder-specific interventions (i.e., exposure and response prevention for OCD, and interoceptive exposure for panic disorders). Ours and earlier findings thus suggest that influencing thoughts and beliefs about exposure therapies may positively affect their use. To foster their dissemination, we need to improve the way exposure is ‘marketed’. Accordingly, it was found that therapists who score high on anxiety sensitivity and endorse negative beliefs about exposure therapy were more inclined to withhold their clients from these types of treatment (Deacon et al. 2013; Meyer et al. 2014). Therapists should therefore be made aware of their misconceptions about the treatment, including their own sensitivity to anxiety, as these factors most likely attenuate treatment outcome (Farrel et al. 2013). However, in our data, avoidance of exposure because it is too challenging or hazardous, did not correlate with its (under) use to any significant degree. Given our efforts to avoid exposure therapy being described as ‘empirically supported’, we expected to limit response bias in terms of over reporting on usage and the appraisal of exposure therapy. Nevertheless, we cannot rule out that therapists in our sample gave answers that were social desirable, so our results should be interpreted with care.

A salient finding was the reported deficit in the availability of exposure-supporting materials at the workplace (e.g., protocols, audio/video equipment, animals), which practical barriers were negatively related to the use of exposure. It is therefore recommended that employers provide sufficient means to facilitate the practice of exposure, while also therapists and group practices are well-advised to make resources available to colleagues, for instance in terms of sharing dedicated video and audio material, and information on facilities where animals can be procured. Our data also showed that therapists who had received more dedicated training in exposure techniques reported fewer such barriers, indicating that additional instruction and training might also help the dissemination of exposure therapies.

With 60 % of the respondents rating their postgraduate training as sufficient, there is much room for improvement in terms of education. As expected, the more highly trained and the more experienced therapists were in exposure techniques, the more they applied these interventions, and the more highly trained therapists were, the higher their affinity with the treatment was. Notably, treatment experience and caseload did not correlate with therapists’ attitudes, suggesting that it is education rather than experience that promotes new insights.

## Conclusions

On the whole, our survey shows that there is some cause for optimism. In the Netherlands most cognitive behavioural therapists have a positive stance on exposure, frequently opt for exposure-based interventions when treating anxiety disorders, and are adequately trained in pertinent techniques. However, as our survey does not clarify whether exposure interventions are delivered correctly or which protocols are adhered to, these are important topics for further research.

Our findings do afford directions for future research and ways to improve the dissemination of exposure treatments. We found that patients with an anxiety disorder not always received the most efficacious, guideline-recommended treatment, even when being treated by a registered cognitive behavioural therapist. About 22 % of patients with a panic disorder were, for instance, rarely offered interoceptive exposure or in vivo exposure exercises. However, this does not mean to say that these patients were treated inappropriately or ineffectively. Moreover, our frequency data revealed that cognitive interventions were amply applied and these may show some degree of overlap with exposure techniques. Interoceptive exposure may then have been used within the framework of a behavioural task and was consequently marked as a cognitive intervention. Also, therapists may have opted for EMDR or ACT (Acceptance and Commitment Training) with particularly anxious patients, given that they reported nearly one fourth of their patients as being unwilling to undergo exposure treatment. To gain a better insight into these matters, future studies should probe more exhaustively which alternatives to exposure interventions are being offered and how this relates to patients’ preferences. Furthermore, these issues strongly relate to the fact that the concrete application of exposure techniques over the therapeutic process could not be reliably captured in our study. As a result, the high use of exposure by a respondent cannot be interpreted as a reflection of providing “adequate treatment”. To chart the state of exposure dissemination more thoroughly, future studies should therefore focus on other types of measurement, e.g. the proportion of exposure interventions used relative to the total treatment process (Külz et al. 2010).

The dissemination of exposure treatments will likely benefit from new approaches to education and training, fostering a more positive attitude toward the treatment itself and its implementation in daily practice. Although the greater majority of our respondents reported an overall satisfaction with their education, 35 % of the certified therapists and 23 % of the supervisors indicated a need for more dedicated instruction. This could have to do with the fact that exposure education was mainly denoted as ‘general’ and to a lesser extent aimed at (disorder-) specific treatments (e.g., instruction on exposure and response prevention for OCD). Because of the relatively large scope of exposure techniques, specific skills and knowledge may need to be given closer attention, although it is unclear how this can be most (cost-) effectively implemented in today’s postgraduate educational system. Our survey also revealed a need for more empirical and diagnostic knowledge. A pilot study comparing training methods for exposure therapies showed that online training was effective and that adding motivation training had the further benefit of increasing positive attitudes toward exposure (Harned et al. 2010). These findings support developments in blended learning (Cucciare et al. 2008), a multimodal approach to education. Effective strategies combine the use of software applications, web-based and live e-learning with classroom education and different methods of self-study. To further the implementation of exposure interventions in clinical practice, future research in this field will need to establish which combination of learning strategies is best suited to train psychologists in the rationale and potential of this effective approach to the treatment of anxiety disorders.

## Abbreviations

ACT: Acceptance and Commitment training
ABCT: Association for Behavioral and Cognitive therapies
CBT: Cognitive Behavioural Therapy
EMDR: Eye Movement Desensitization and Reprocessing
EST: Empirically supported treatment
NICE: National Institute for Health and Clinical Excellence
OCD: Obsessive compulsive disorder
PTSD: Posttraumatic stress disorder
SPSS: Statistical Package for the Social Sciences
VGCt: Dutch Association for Behavioural and Cognitive Therapy

## References

[CR1] Abramowitz JS (1996). Variants of exposure and response prevention in the treatment of obsessive-compulsive disorder: A meta-analysis. Behavior Therapy.

[CR2] Becker CB, Zayfert C, Anderson E (2004). A survey of psychologists’ attitudes towards and utilization of exposure therapy for PTSD. Behaviour Research and Therapy.

[CR3] Bradley R, Greene J, Russ E, Dutra L, Westen D (2005). A multidimensional meta-analysis of psychotherapy for PTSD. American Journal of Psychiatry.

[CR4] Cahill SP, Rothbaum BO, Rosick PA, Folette V, Foa EB, Keane TM, Friedman MJ, Cohen JA (2009). Cognitive-behavioural therapy for adults. Effective treatments for PTSD.

[CR5] Craske MG (1999). Anxiety disorders. Psychological approaches to theory and treatment.

[CR6] Cucciare MA, Weingardt KR, Villafranca S (2008). Using blended learning to implement evidence-based psychotherapies. Clinical Psychology: Science and Practice.

[CR7] Deacon BJ, Farrel NR, Kemp JJ, Dixon LJ, Sy JT, Zhang AR, McGrath PB (2013). Assessing therapist reservations about exposure therapy for anxiety disorders: The Therapist Beliefs about Exposure Scale. Journal of Anxiety Disorders.

[CR8] Farrel NR, Deacon BJ, Dixon LJ, Lickel JJ (2013). Theory-based training strategies for modifying practitioner concerns about exposure therapy. Journal of Anxiety Disorders.

[CR9] Fedoroff IC, Taylor S (2001). Psychological and pharmacological treatments of social phobia: A meta-analysis. Journal of Clinical Psychopharmacology.

[CR10] Feeny NC, Hembree EA, Zoellner LA (2003). Myths regarding exposure therapy for PTSD. Cognitive and Behavioral Practice.

[CR11] Feske U, Chambless DL (1995). Cognitive behavioral versus exposure only treatment for social phobia: A meta-analysis. Behavior Therapy.

[CR12] Freiheit SR, Vye C, Swan R, Cady M (2004). Cognitive-behavioral therapy for anxiety: Is dissemination working?. Behavior Therapist.

[CR13] Goisman RM, Warshaw MG, Keller MB (1999). Psychosocial treatments prescriptions for generalized anxiety disorder, panic disorder, and social phobia, 1991–1996. American Journal of Psychiatry.

[CR14] Gould RA, Otto MW, Pollack MH, Yap L (1997). Cognitive behavioral and pharmacological treatment of generalized anxiety disorder: a preliminary meta-analysis. Behavior Therapy.

[CR15] Harned MS, Dimeff LA, Woodcock EA, Skutch JM (2010). Overcoming barriers to disseminating exposure therapies for anxiety disorders: A pilot randomized controlled trial of training methods. Journal of Anxiety Disorders.

[CR16] Külz KA, Hassenpflug K, Riemann D, Linster HW, Dornberg M, Voderholzer U (2010). Psychotherapeutic Care in OCD Outpatients – Results from an Anonymous Therapist Survey. Psychotherapie Psychosomatik Medizinische Psychologie (PPMP).

[CR17] LSMR (Dutch National Steering-Group Multidisciplinary Guideline Development for Mental Healthcare) (2009). Revision of the multidisciplinary guideline. Update anxiety disorders (first revision).

[CR18] LSMR (Dutch National Steering-Group Multidisciplinary Guideline Development for Mental Healthcare) (2013). Guideline for diagnostics, treatment and guidance of adult patients with an anxiety disorder (third revision).

[CR19] Marcks BA, Weisberg RB, Keller MB (2009). Psychiatric treatment received by primary care patients with panic disorder with and without agoraphobia. Psychiatric Services.

[CR20] McAleavy AA, Castonguay LG, Goldfried MR (2014). Clinical Experiences in Conducting Cognitive-Behavioral Therapy for Social Phobia. Behavior Therapy.

[CR21] Meyer JM, Farrell NR, Kemp JJ, Blakey SM, Deacon BJ (2014). Why do clinicians exclude anxious clients from exposure therapy?. Behaviour Research and Therapy.

[CR22] National Institute for Health and Clinical Excellence (2011). Generalized anxiety disorder and panic disorder (with or without agoraphobia) in adults: Management in primary, secondary and community care. CG113.

[CR23] Olatunji BO, Deacon BJ, Abramowitz JS (2009). The cruelest cure? Ethical issues in the implementation of exposure-based treatments. Cognitive and Behavioral Practice.

[CR24] Richard DCS, Gloster AT, Richard DCS, Lauterbach D (2007). Exposure therapy has a public relations problem: A dearth of litigation amid a wealth of concern. Handbook of exposure therapies.

[CR25] Rosa-Alcázar AI, Sánchez-Meca J, Gómez-Conesa A, Marín-Martínez F (2008). Psychological treatment of obsessive-compulsive disorder: A meta-analysis. Clinical Psychology Review.

[CR26] Sánchez-Meca J, Rosa-Alcázar AI, Marín-Martínez F, Gómez-Conesa A (2010). Psychological treatment of panic disorder with or without agoraphobia: A meta-analysis. Clinical Psychology Review.

[CR27] Shafran R, Clark DM, Fairburn CG, Arntz A, Barlow DH, Ehlers A, Freeston M, Garety PA, Hollon SD, Ost LG, Salkovskis PM, Williams JMG, Wilson GT (2009). Mind the gap: Improving the dissemination of CBT. Behaviour Research and Therapy.

[CR28] van Balkom AJ, Bakker A, Spinhoven P, Blaauw BM, Smeenk S, Ruesink B (1997). A meta-analysis of the treatment of panic disorder with or without agoraphobia: a comparison of psychopharmacological, cognitive-behavioral, and combination treatments. Journal of Nervous and Mental Disease.

[CR29] van Minnen A, Hendriks H, Olff M (2010). When do trauma experts choose exposure therapy for PTSD patients? A controlled study of therapist and patient factors. Behaviour Research and Therapy.

[CR30] Weissman MM, Verdeli H, Gameroff MJ, Bledsoe SE, Betts K, Mufson L, Fitterling H, Wickramaratne P (2006). National survey of psychotherapy training in psychiatry, psychology, and social work. Archives of General Psychiatry.

[CR31] Wolf AW, Goldfried MR (2014). Clinical Experiences in Using Cognitive-Behavior Therapy to Treat Panic Disorder. Behavior Therapy.

[CR32] Wolitzky-Taylora KB, Horowitz JD, Powers MB, Telch MJ (2008). Psychological approaches in the treatment of specific phobias: A meta-analysis. Clinical Psychology Review.

